# Influence of 5 major Salmonella pathogenicity islands on NK cell depletion in mice infected with *Salmonella enterica *serovar Enteritidis

**DOI:** 10.1186/1471-2180-10-75

**Published:** 2010-03-12

**Authors:** Daniela Karasova, Alena Sebkova, Hana Havlickova, Frantisek Sisak, Jiri Volf, Martin Faldyna, Petra Ondrackova, Vladimir Kummer, Ivan Rychlik

**Affiliations:** 1 Veterinary Research Institute, Hudcova 70, 621 00 Brno, Czech Republic

## Abstract

**Background:**

In this study we were interested in the colonisation and early immune response of Balb/C mice to infection with *Salmonella *Enteritidis and isogenic pathogenicity island free mutants.

**Results:**

The virulence of *S*. Enteritidis for Balb/C mice was exclusively dependent on intact SPI-2. Infections with any of the mutants harbouring SPI-2 (including the mutant in which we left only SPI-2 but removed SPI-1, SPI-3, SPI-4 and SPI-5) resulted in fatalities, liver injures and NK cell depletion from the spleen. The infection was of minimal influence on counts of splenic CD4 CD8 T lymphocytes and γδ T-lymphocytes although a reduced ability of splenic lymphocytes to respond to non-specific mitogens indicated general immunosuppression in mice infected with SPI-2 positive *S*. Enteritidis mutants. Further investigations showed that NK cells were depleted also in blood but not in the caecal lamina propria. However, NK cell depletion was not directly associated with the presence of SPI-2 and was rather an indicator of virulence or avirulence of a particular mutant because the depletion was not observed in mice infected with other attenuated mutants such as *lon *and *rfaL*.

**Conclusions:**

The virulence of *S*. Enteritidis for Balb/C mice is exclusively dependent on the presence of SPI-2 in its genome, and a major hallmark of the infection in terms of early changes in lymphocyte populations is the depletion of NK cells in spleen and blood. The decrease of NK cells in circulation can be used as a marker of attenuation of *S*. Enteritidis mutants for Balb/C mice.

## Background

*Salmonella enterica *serovar Enteritidis (*S*. Enteritidis) is one of the major causative agents of human food borne diseases. Besides humans, *S*. Enteritidis is frequently associated with poultry but may be isolated also from pigs, cattle as well as different reptiles. If mice are infected experimentally, especially the highly susceptible Nramp-defective Balb/C lineage, *S*. Enteritidis causes a disease similar to that caused by *S*. Typhimurium. To successfully colonise such a broad range of different hosts, *S*. Enteritidis has acquired genes which are frequently clustered at particular parts of chromosome called *Salmonella *Pathogenicity Islands (SPI). Although there are up to 14 different pathogenicity islands, the presence of which varies among different serovars of *Salmonella enterica *(*S. enterica*), 5 of these can be found in all *S. enterica *serovars.

The SPI-1 and SPI-2 pathogenicity islands are considered as the most important for *S. enterica *virulence. Proteins encoded by SPI-1 form a type III secretion system (TTSS) which mediates the translocation of *S. enterica *proteins into a host cell across its cytoplasmic membrane. The translocated proteins induce cytoskeletal rearrangements which results in *S. enterica *uptake even by non-professional phagocytes [1,2]. Genes localised within SPI-2 encode proteins of another TTSS expressed by *S. enterica *inside host cells where it translocates its proteins across the phagosomal membrane and increases intracellular survival [3]. The functions of the genes localised on the remaining SPIs are less well characterised; for SPI-3 genes conflicting information has been published suggesting their role both in gut colonisation and intracellular survival [4,5]. SPI-4 genes are required for the intestinal phase of disease [5] although a SPI-4 requirement for systemic infection of mice has been also reported [6]. Genes localised at SPI-5 are co-regulated with either SPI-1 or SPI-2 genes and therefore represent a dually controlled system [7,8].

After oral ingestion, *S. enterica *comes into contact with the intestinal epithelial lining and using the SPI-1 encoded TTSS it enters M-cells and enterocytes. After crossing the epithelium *S. enterica *interacts with neutrophils and macrophages. The result of these initial events is critical for the outcome of the disease. If *S. enterica *is not recognised by host cells, and the proinflammatory immune response in the gut is not induced, it is likely that the infection will develop into a typhoid-like disease [9-11]. During the course of the typhoid-like infection of mice, *S. enterica *colonises internal organs such as liver and spleen where it is found in macrophages, neutrophils, and T- and B-lymphocytes [12]. Why the immune system of a host does not respond properly to *S. enterica *infection during the typhoid disease has never been explained in sufficient detail although it is known that *S. enterica *is capable of induction of apoptosis in macrophages [13,14], inhibition of antigen presentation by dendritic cells [15] and also NK cell depletion [16].

Except for the role of SPI-1 in invasiveness the non-professional phagocytes and SPI-2 in intracellular survival, roles of the remaining 3 major pathogenicity islands in the interactions of *S. enterica *with host immune system are not too much elucidated. In this study we therefore removed the 5 major pathogenicity islands from the *S*. Enteritidis genome in a step-by-step manner and used such mutants for oral infection of Balb/C mice. We found out that virulence in mice was exclusively dependent on SPI-2 because even the mutant in which SPI-1, SPI-3, SPI-4 and SPI-5 pathogenicity islands had been removed from its genome was as virulent as the wild type strain. When the changes in splenic lymphocytes were determined 5 days post infection, B-lymphocytes, CD8 and γδ T-lymphocytes did not change regardless of the mutant used for the infection. The only lymphocyte population which decreased in the spleen and blood after the infection with virulent *S*. Enteritidis, but not the attenuated mutants, was formed by NK cells.

## Results

**Figure 1:**
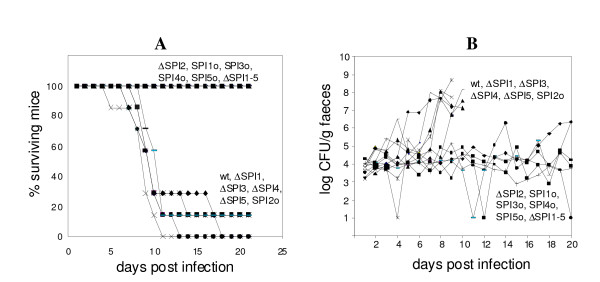
**Death rates (panel A) and faecal shedding (panel B) in mice orally infected with *S*. Enteritidis and SPI mutants**. Mice infected with SPI-2 positive mutants exhibited high faecal shedding and died within 3 weeks post-infection. Faecal shedding of individual mice which survived the infection with ΔSPI1, ΔSPI4 and SPI2o (i.e. SPI-2 positive mutants) beyond day 10 is not shown for clarity. Survival rates of the mice infected with ΔSPI2, ΔSPI1-5 and SPI1o, SPI3o, SPI4o and SPI5o were significantly different from those infected with the wild type *S*. Enteritidis as determined by Logrank test at P < 0.01.

**Figure 2:**
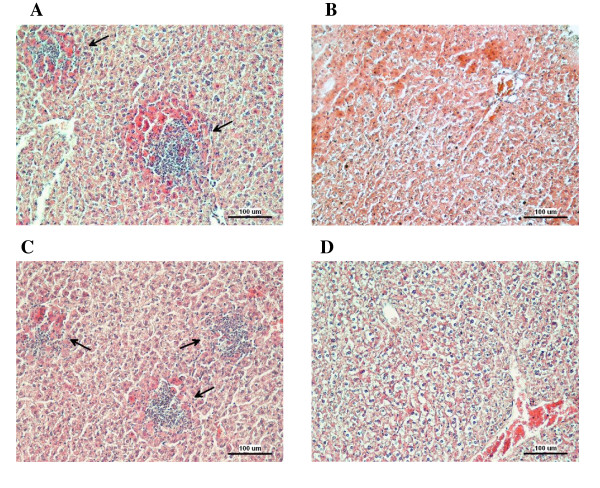
**Histological analysis of liver samples of mice infected with the wild-type *S*. Enteritidis or SPI-2 mutants**. Arrows points towards necrotic areas with neutrophil infiltration. A - liver of mice infected with the wild type *S*. Enteritidis, B - liver of mice infected with the ΔSPI2 mutant, C - liver of mice infected with the SPI2o mutant, D - liver of mice infected with the ΔSPI1-5 mutant. Exactly the same pathology, depending on the presence or absence of SPI-2, was observed in the other mice infected with the other SPI mutants. Bar indicates 100 μm.

Mice infected with the wild-type *S*. Enteritidis or any of the mutants harboring SPI-2 died within 3 weeks post-infection whereas all mice infected with any of the mutants not possessing SPI-2 survived the infection (Figure 1). Mice infected with mutants harboring SPI-2 in their genome exhibited high counts of *S*. Enteritidis in liver and spleen at day 5 post infection (Table 1). Histological examination did not reveal any difference in the caecum in the animals while necrotic foci were observed in the livers of mice infected with the wild type *S*. Enteritidis or the mutants harboring SPI-2 (Figure 2). As a result of these observations, in some of the data analyses described below, we clustered the strains into two groups, SPI-2 positive and SPI-2 negative, regardless of the presence or absence of additional pathogenicity islands.

**Table 1:** Counts of *S*. Enteritidis in liver, spleen and caecum 5 days post oral infection.

	liver	spleen	caecum
	(log CFU/g of tissue)		
wt	4.97 ± 2.22	5.52 ± 2.47	4.19 ± 2.49
ΔSPI1	5.10 ± 1.12	5.79 ± 1.07	4.18 ± 1.15
ΔSPI2	0.25 ± 0.43*	0.56 ± 0.50*	2.05 ± 1.49
ΔSPI3	5.13 ± 0.19	6.19 ± 0.36	3.79 ± 0.02
ΔSPI4	4.83 ± 1.42	5.60 ± 1.36	2.97 ± 1.75
ΔSPI5	3.52 ± 1.79	4.29 ± 2.32	2.90 ± 1.40
SPI1o	0.33 ± 0.47*	0.33 ± 0.47*	2.20 ± 1.59
SPI2o	5.29 ± 0.87	5.82 ± 1.07	3.76 ± 0.77
SPI3o	0.00 ± 0.00*	0.00 ± 0.00*	0.33 ± 0.47*
SPI4o	0.00 ± 0.00*	0.00 ± 0.00*	1.68 ± 2.38
SPI5o	0.00 ± 0.00*	0.00 ± 0.00*	1.07 ± 1.51*
ΔSPI1-5	0.00 ± 0.00*	0.00 ± 0.00*	1.06 ± 1.50*

* T-test different at P < 0.01 from the mice infected with the wild type *S*. Enteritidis

Next we were interested to what extent the different virulence of individual SPI mutants would be reflected in host immune responses. Since the cell-mediated immune response is important for the protection against *S. enterica *and lymphocytes play an important role in the co-ordination of the host's immune response, we first characterised changes in lymphocyte subpopulations after the infection with the wild type strain and all the SPI mutants. When 3 mice from each group were sacrificed on day 5 post infection i.e. before the onset of fatalities, no differences in the distribution of splenic B-lymphocytes, CD3 T-lymphocytes and γδ T-lymphocytes were observed. Of the Th lymphocytes, the only statistically significant change was the decrease in number of CD4 lymphocytes observed after the infection with the SPI2o mutant when compared with the non-infected mice. CD4 lymphocytes also decreased in number after the infection with the ΔSPI1 mutant, i.e. another mutant in which, similarly to the SPI2o mutant, SPI-1 was absent while SPI-2 was present, although in this case the decrease did not reach statistical significance (P = 0.0634, see also Table 2). However, we did not investigate this further because we found another lymphocyte subpopulation which exhibited a more pronounced changes which also correlated with the severity of the infection (see below).

**Table 2:** Two-colour flow cytometry of splenic CD3 and CD19 T- and B-lymphocytes, CD4 and CD8 T-lymphocytes, and γδ T-lymphocytes in mice infected with *S*. Enteritidis 5 days post-infection.

	CD3+19-	CD19+3-	CD19-3-	CD4+8+	CD4+8-	CD4-8+	γδT
wt	52.5 ± 0.76	41.8 ± 2.07	5.4 ± 1.33*	1.4 ± 0.33	31.1 ± 2.93	14.8 ± 0.28	0.93 ± 0.09
ΔSPI1	57.1 ± 6.50	39.6 ± 5.80	3.2 ± 0.98*	0.9 ± 0.10	24.5 ± 6.26^&^	16.2 ± 2.05	0.50 ± 0.08
ΔSPI2	50.3 ± 3.77	41.8 ± 2.91	7.7 ± 0.98	1.1 ± 0.13	27.4 ± 1.80	14.5 ± 0.35	0.80 ± 0.22
ΔSPI3	55.1 ± 3.26	40.9 ± 4.37	3.9 ± 1.59*	1.4 ± 0.33	27.1 ± 4.63	16.4 ± 1.19	0.53 ± 0.05
ΔSPI4	56.5 ± 4.24	39.5 ± 3.61	4.1 ± 1.42*	1.3 ± 0.46	26.8 ± 2.80	16.2 ± 1.05	0.67 ± 0.24
ΔSPI5	60.1 ± 5.22	35.8 ± 4.05	3.9 ± 1.25*	1.1 ± 0.21	33.8 ± 1.01	14.9 ± 1.33	0.57 ± 0.05
ΔSPI1-5	55.5 ± 3.07	36.4 ± 2.86	8.0 ± 1.79	2.1 ± 0.41	34.6 ± 3.01	17.2 ± 0.26	0.70 ± 0.08
SPI1 only	55.6 ± 3.78	37.4 ± 2.54	7.1 ± 1.75	0.9 ± 0.26	35.0 ± 3.44	16.1 ± 0.70	0.97 ± 0.05
SPI2 only	43.0 ± 2.50	49.0 ± 6.63	3.8 ± 2.02*	0.6 ± 0.25	23.8 ± 5.80*	14.4 ± 1.16	0.80 ± 0.22
SPI3 only	62.7 ± 4.28	29.9 ± 4.46	7.5 ± 0.49	1.4 ± 0.05	29.2 ± 2.92	18.6 ± 0.87	0.80 ± 0.16
SPI4 only	64.2 ± 4.33	28.9 ± 3.60	7.0 ± 0.72	1.6 ± 0.38	33.6 ± 4.07	18.9 ± 1.94	0.73 ± 0.05
SPI5 only	57.8 ± 0.99	35.5 ± 1.54	6.7 ± 1.04	1.4 ± 0.01	34.6 ± 0.49	17.0 ± 1.11	1.07 ± 0.05
non infect	53.0 ± 10.00	39.2 ± 10.54	7.7 ± 1.12	1.2 ± 0.44	33.7 ± 6.01	14.4 ± 2.55	1.01 ± 0.32

Numbers show average percentage ± standard deviation out of total CD45 positive lymphocytes. * T-test different at P < 0.05 from the non-infected mice, ^&^P = 0.0634.

**Figure 3:**
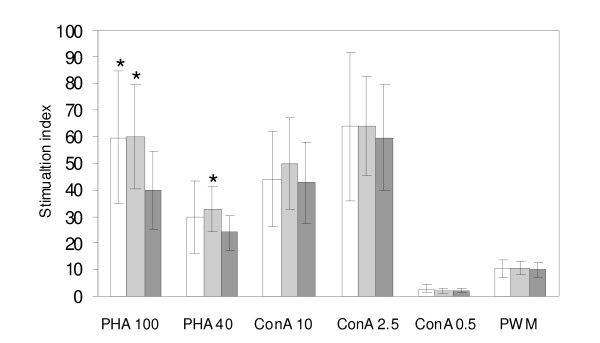
**Lymphocyte proliferation assay from non-infected mice (white columns), and mice infected with SPI2-negative (light grey columns) and SPI2-positive (dark grey columns) *S*. Enteritidis mutants after the stimulation with different concentrations of phytohaemagglutinin (PHA), concanavalin A (ConA) or pokeweed mitogen (PWM)**. * - t-test different from the mice infected with the SPI-2 positive *S*. Enteritidis at P < 0.05.

Although the T- and B-lymphocytes did not change in their relative counts in the spleens of infected mice, we observed that the lymphocytes from mice infected with SPI-2 positive mutants were suppressed in their response to non-specific mitogens. Due to the limited number of mice in individual groups this difference was not significant when individual groups of mice were compared with the non-infected controls. However, when the mice were grouped according to their virulence i.e. according to the presence or absence of SPI-2, all SPI-2 positive virulent strains induced significant immunosuppression when stimulated by phytohemagglutinin but not the other two mitogens tested (Figure 3).

**Figure 4:**
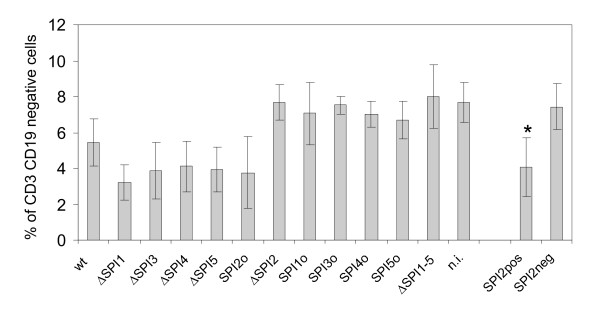
**CD3 CD19 double-negative lymphocytes in spleens of mice infected with *S*. Enteritidis SPI mutants; n.i. - non-infected mice**. The Y-axis shows percentage of CD3 CD19 double-negative lymphocytes out of total CD45 positive splenic lymphocytes. Columns SPI2pos and SPI2neg show average values for all mice clustered into two groups according to being infected with either any of the SPI-2 positive or the SPI-2 negative mutants. * - t-test different from SPI2neg at P < 0.01.

**Figure 5:**
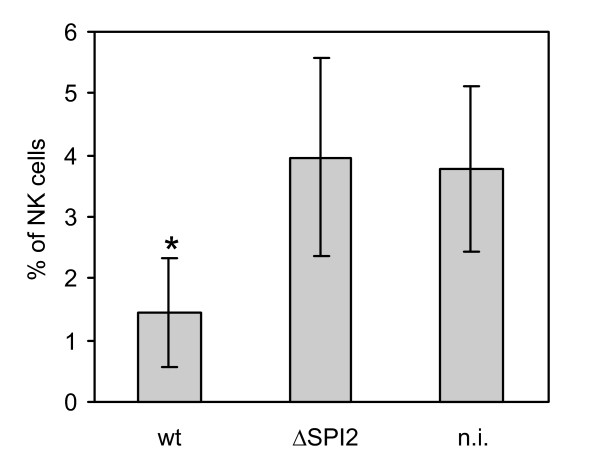
**Presence of NK cells in spleen of mice 5 days post infection with the wild type *S*. Enteritidis (wt) or ΔSPI2 mutant averaged from the animal infections 2, 3 and 4**. n.i. - non-infected mice. * - t-test different from the non-infected or ΔSPI2 mutant infected mice at P < 0.01.

The lymphocyte subpopulation which exhibited the most pronounced changes and which also corresponded with the severity of infection was formed by the CD3 CD19 double negative lymphocytes (Table 2 and Figure 4). The numbers of these cells decreased in the spleens of mice which would normally go on to succumb to the infection i.e. in mice infected with the wild type *S*. Enteritidis or any mutant with an intact SPI-2. The CD3 CD19 double negative lymphocytes could be formed either by monocytes gated together with the lymphocytes, or the NK cells. To distinguish between these two potential cell populations, additional experiments were performed. In this case, mice were infected only with the wild type *S*. Enteritidis and ΔSPI2 mutant, and using four-color flow cytometry CD19, CD3 double negative lymphocytes were further characterised according to the presence or absence of CD14 and CD16. The dominant part of the CD3 CD19 double negative population constituted of CD16+ CD14- cells and these were the cells which decreased after the infection with virulent *S*. Enteritidis. Since CD3 CD14 CD19 negativity and CD16 positivity is characteristic for the NK cells, we concluded that the infection with the wild type strain or any mutant of *S*. Enteritidis with functional SPI-2 resulted in the depletion of NK cells in spleen (Figure 5).

**Figure 6:**
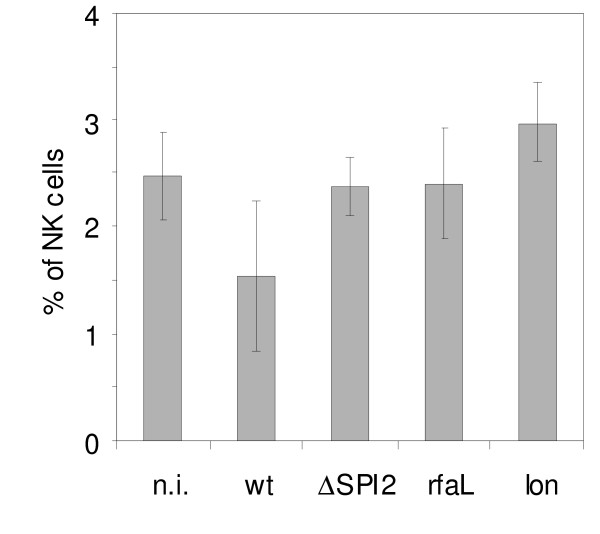
**Presence of NK cells in spleen of mice 5 days post infection with the wild type *S*. Enteritidis (wt) or attenuated ΔSPI2, *rfaL *or *lon *mutants as determined in the animal infection 2. n.i. - non-infected mice**. The NK cells depletion was not specific for the ΔSPI2 mutant but was a general indicator of the mutant's virulence or avirulence. Since there were only 3 animals per group and greater variation was observed among the mice infected with the wild type *S*. Enteritidis, none of the differences in this experiment reached statistical significance.

Next we investigated whether the depletion of NK cells was associated specifically with the presence of SPI-2 or whether it was rather a general indicator of *S*. Enteritidis virulence. In this experiment we infected mice with another two attenuated mutants with defects in *lon *or *rfaL *and monitored NK cells in the spleen of infected mice. As shown in Figure 6, the NK cells decreased in the spleen only after the infection with the wild type *S*. Enteritidis, but not after the infection with the *rfaL *or *lon *mutants.

Although it was obvious that the NK cells decreased after infection with the wild type strain or virulent mutants, the reason for the NK cell depletion was unknown. We considered two alternative hypotheses - either the NK cells migrated out of the spleen possibly to the intestinal tract or they died as a result of the extensive killing of *S*. Enteritidis-positive splenocytes. To test these hypotheses, we performed two additional experiments. In the first experiment we analysed cytokine signaling in the intestinal tract of the infected mice and in the second experiment we determined NK cell counts not only in the spleen but also in blood and the caecal lamina propria. These experiments were performed only with the wild type *S*. Enteritidis and ΔSPI2 mutant.

**Figure 7:**
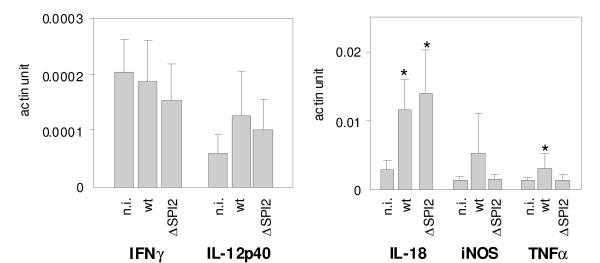
**Cytokine response in caecum of mice infected with the wild type *S*. Enteritidis (wt) and ΔSPI2 mutant. n.i. - non-infected mice**. * - t-test different from the non-infected mice at P < 0.05.

When the cytokine signaling in the ceacal samples was determined, we did not find any differences in the expression of IFNγ, iNOS and IL-12p40. Numerically low, but statistically significant induction of TNFα was observed in caecum of mice infected with the wild type *S*. Enteritidis. Mice also responded to *S*. Enteritidis infection by the upregulation of IL-18 although this cytokine was significantly upregulated in mice infected both by the wild type *S*. Enteritidis and the ΔSPI2 mutant (Figure 7).

**Figure 8:**
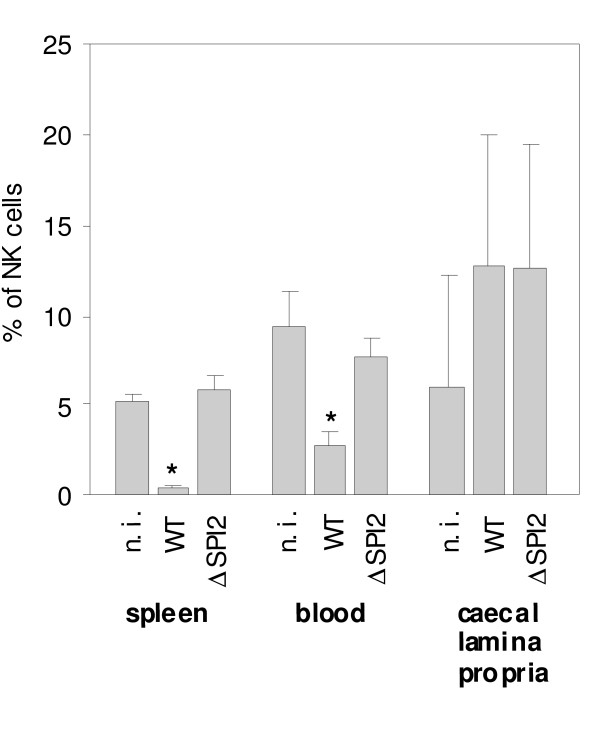
**Distribution of NK cells in spleen, blood and caecal lamina propria of mice infected with the wild type *S*. Enteritidis (wt) and ΔSPI2 mutant as determined in the animal infection 4**. n.i. - non-infected mice. * - t-test different from the non-infected mice at P < 0.01.

Finally we tested whether the depletion of NK cells could be caused by their migration to the caecal lamina propria. We therefore infected mice with wild type *S*. Enteritidis and ΔSPI2 mutant, and besides the spleen we also determined the counts of the NK cells in blood and the lamina propria. In blood, a significant decrease in NK cells post wild-type *S*. Enteritidis infection was observed. In the lamina propria, the numerical increase in NK cells was observed although this increase did not reach statistical significance (Figure 8).

## Discussion

Similar to the observations of others, progress of the infection in mice, characterised by fecal shedding, fatalities, liver and spleen colonisation and liver injury, was dependent on the presence of SPI-2 but not any other SPI [3,17,18]. The exclusivity of SPI-2 dependence for *S*. Enteritidis virulence for mice was such that even in the absence of all remaining SPIs, i.e. in the case of SPI2o mutant, this mutant was capable of causing typhoid similar to that caused by the wild-type strain. This observation was slightly unexpected for the mutants without SPI-1. However Murray and Lee already reported on minimal influence of the removal of the whole SPI-1 on the virulence of *S*. Typhimurium for Balb/C mice [18] and also single gene mutants in *sopB*, *sopD *or *sipA *were only weakly attenuated [19,20] or the attenuation was expressed only as a minor delay in mean time to death [21]. In addition, dose dependent difference in the virulence of *sopB *mutant of *S*. Typhimurium was described [20] and since we used only a single dose corresponding to 100× LD_50_, minor phenotypic differences associated with the presence or absence of SPI-1 could remain undetected.

The infection did not influence the counts of T- and B-lymphocytes in the spleen at the time of sampling, similarly to the findings of Geddes et al [12]. We did not even observe an increase in γδ T-lymphocytes although these were reported to increase in mice after infection with a virulence plasmid-cured derivative of *S*. Choleraesuis [22]. Although there were no changes in these cell populations, general immunosuppression has been observed when PHA was used as the mitogen for stimulation. Since the immunosuppression was not observed when ConA and PHW were used for the stimulation, it can be expected that the population which was primarily immunosuppressed was that represented by the CD4 Th lymphocytes [23].

The most significant changes correlating with the severity of infection were observed in NK cells that could result in the reduced production of pro-inflammatory IFNγ [24,25] and immunosuppressive IL-10 [26]. The decrease in NK cells in systemic sites may result also in a decrease in Th1 polarisation of the immune response [27] followed by mice fatalities. The depletion of NK cells in mice after the infection with wild-type *Salmonella *has been previously described [16]. However, whether the virulence mechanisms encoded by any of the pathogenicity islands are involved in this response has never been addressed. Our results indicate that there is no direct correlation between the presence of any of the SPIs and the NK cell depletion. Although the decrease in NK cell counts was not observed in all mice infected with SPI2-negative *S*. Enteritidis, it was also not observed in mice infected with the attenuated *S*. Enteritidis mutants defective in *lon *or *rfaL*. The depletion of NK cells therefore does not appear to be directly influenced by the SPI-2 encoded type III secretion system and instead, it seems to be a general indicator of virulence or attenuation of a mutant for mice.

Finally we considered whether the depletion of NK cells in spleen was caused by the migration of these cells from the spleen to other tissues such as those in the intestinal tract since the accumulation of NK cell in the intestinal tract, although in a slightly different model of streptomycin-treated mice, has been reported [24]. The decrease of NK cells in spleen and circulation together with a minor increase of NK cells in caecum (Figure 8) would support the hypothesis on migration. However, because the NK cell increase in the lamina propria as well as the cytokine response in caecum was numerically similar in mice infected with the wild-type *S*. Enteritidis and the ΔSPI2 mutant, while the NK cell depletion in spleen and blood occurred only after the infection with the wild type *S*. Enteritidis, the decrease in NK cells in spleen and circulation cannot be directly linked with their migration to caecum.

## Conclusions

In this study we have shown that the virulence of *S*. Enteritidis for Balb/C mice is exclusively dependent on the presence of SPI-2 in its genome, and a major hallmark of the infection in terms of changes in lymphocyte populations is the depletion of NK cells in the spleen and circulating blood. The decrease of NK cells in circulation can be used as a marker of attenuation or virulence of different *S*. Enteritidis mutants for Balb/C mice.

## Methods

### Bacterial strains and growth conditions

*S*. Enteritidis147, a clone resistant to nalidixic acid, was used in this study [28]. Isogenic mutants without individual SPIs (SPI-1 to SPI-5), *lon *and *rfaL *mutants are listed in Table 3. SPI mutants were generated by a modified procedure of λ Red recombination [29] which we have described previously [30]. Absence of individual SPIs was confirmed by positive PCR using primers flanking individual SPIs and negative PCR using primers specific for the genes localised inside each of the pathogenicity island (not shown). The strains were propagated in LB broth or LB agar at 37°C.

**Table 3:** List of strains used in this study.

strain	strain ID	SPI present	SPI absent	reference
*S*. Enteritidis 147 Nal wild type	7F4	1, 2, 3, 4, 5	none	[28]
*S*. Enteritidis 147 Nal ΔSPI1	4A10	2,3,4,5	1	[30]
*S*. Enteritidis 147 Nal ΔSPI2	5D10	1,3,4,5	2	[30]
*S*. Enteritidis 147 Nal ΔSPI3	6A9	1,2,4,5	3	[30]
*S*. Enteritidis 147 Nal ΔSPI4	4B10	1,2,3,5	4	[30]
*S*. Enteritidis 147 Nal ΔSPI5	4J1	1,2,3,4	5	[30]
*S*. Enteritidis 147 Nal ΔSPI1-5	5E9	none	1,2,3,4,5	[30]
*S*. Enteritidis 147 Nal SPI1o	5G10	1	2,3,4,5	[30]
*S*. Enteritidis 147 Nal SPI2o	5H9	2	1,3,4,5	[30]
*S*. Enteritidis 147 Nal SPI3o	5J10	3	1,2,4,5	[30]
*S*. Enteritidis 147 Nal SPI4o	5D9	4	1,2,3,5	[30]
*S*. Enteritidis 147 Nal SPI5o	5H10	5	1,2,3,4	[30]
*S*. Enteritidis 147 Nal Δlon	16H2	1, 2, 3, 4, 5	none	[33]
*S*. Enteritidis 147 Nal ΔrfaL	14E5	1, 2, 3, 4, 5	none	[33]

### Experimental infection of mice

In all the experiments, six-week-old Balb/C mice were orally infected with 10^4 ^CFU (equivalent to 100 × LD_50 _of the wild type strain) of the wild type strain or each of the mutants in a volume of 0.1 ml using a gastric gavage without any neutralisation of gastric acid prior the infection. In the first animal infection, 12 groups of 10 mice each were infected with all the SPI mutants and wild type *S*. Enteritidis. A negative control group consisted of 3 uninfected animals. On day 5 post-infection, 3 mice from each group including all non-infected control mice were sacrificed and used for the determination of bacterial counts in liver, spleen and caecum, two-color flow cytometry of splenic lymphocytes, histology in liver and caecum, and lymphocyte proliferation assay. The remaining 7 mice were left for monitoring of feacal shedding and mortalities until day 21 post infection when the experiment was terminated. Faecal shedding was monitored on a daily basis by transferring the mice into a clean plastic box and collecting pooled fresh droppings 30 minutes later. Bacterial counts in liver, spleen, caecal content and faecal droppings were determined using a standard plating method described previously [31]. For the purposes of statistical analysis, a viable count of log_10 _< 2.5 (limit for direct plate detection) obtained from a sample positive only after enrichment was rated as log_10 _= 1.0 whereas samples negative for *S*. Enteritidis after enrichment were rated as log_10 _= 0. During the post mortem analysis, liver and caecal samples were also taken for histological examinations. The samples were fixed in 10% neutral buffered formalin for 24 h, embedded in paraffin wax, sectioned at 5 μm, and stained with haematoxylin-eosin.

In the second animal infection, 3 mice per group, including 3 non-infected mice, were infected with the wild-type *S*. Enteritidis, or with ΔSPI2, *lon *or *rfaL *mutants. In this experiment, four-colour flow cytometry detecting CD3, CD19, CD14 and CD16 in splenic lymphocytes was performed.

In the third animal infection, 5 mice per group, including 5 non-infected mice, were infected with the wild type *S*. Enteritidis or ΔSPI2 mutant. In this experiment, four-colour flow cytometry detecting CD3, CD19, CD14 and CD16 in splenic lymphocytes was repeated and in addition, cytokine signaling in caecum has been determined by RT PCR.

In the fourth animal infection, 5 mice per group, including 5 non-infected mice, were infected with the wild type *S*. Enteritidis and ΔSPI2 mutant, and four-colour flow cytometry detecting CD3, CD19, CD14 and CD16 cells in lymphocytes from spleen, blood and caecal lamina propria was performed.

All the animal infections were performed according to the relevant national legislation and were approved and supervised by the institutional Ethics Committee on Animal Experiments followed by the approval of the Animal Welfare Committee at the Ministry of Agriculture of the Czech Republic.

### Lymphocyte proliferation assay

The proliferation activity of lymphocytes was determined using the mitogen-driven proliferation assay. Spleen tissues were collected into RPMI 1640 medium (Sigma, St. Louis, USA) and cell suspensions were prepared by pressing the tissue through a fine nylon mesh. After ammonium chloride-mediated lysis of erythrocytes, the density of the suspension was adjusted to 10^6 ^per ml of RPMI 1640 medium supplemented with 10% pre-colostral calf serum, 100 000 U/l penicillin and 0.2 g/l streptomycin. Two hundred microliters of the cell suspension were transferred in triplicate into the wells of a 96-well flat-bottomed microtitre plate. Mitogens were used as follow: phytohaemagglutinin (PHA) at the concentrations 100 μg/ml and 40 μg/ml, concanavalin A (ConA) at the concentrations 10 μg/ml, 2.5 μg/ml, and 0.5 μg/ml, and pokeweed mitogen (PWM) at the concentration 10 μg/ml. Lymphocytes incubated in the absence of these mitogens served as non-stimulated controls. The microplates were incubated at 37°C under the 5% CO_2 _atmosphere for 3 days, and 20 hours before harvesting (FilterMate Harvestor, Packard Bioscience Instrument Company), 50 μl of medium with ^3^H-thymidine (5 μCi/ml) was added. The incorporation of ^3^H-thymidine was analyzed by a microplate scintillation and luminescence counter (TopCount NXT™, Packard Bioscience Instrument Company). The results were expressed as stimulation indices, which have been calculated as the ratio of counts per minute in stimulated samples and non-stimulated controls.

### Flow cytometry

For the flow cytometry, splenic lymphocytes were purified as described above. Lymphocytes from blood were isolated by the whole-blood lysis technique as described previously [32]. To isolate lymphocytes from gut tissue, the tissue was incubated in HBSS-2 containing 2 mM DTT and 0.5 mM EDTA at 37°C for 40 min followed by collagenase type IV (50 U/ml) treatment for additional 90 min. The lymphocytes were finally isolated from cell suspensions by a gradient centrifugation with 80% Percol.

In the next step, the cells were washed in PBS with 0.2% gelatin from cold water fish skin, 0.1% sodium azide and 0.05 mM EDTA and resuspended in the same buffer to a density of 5 × 10^6 ^cells/ml. The following anti-mouse monoclonal antibodies directed against surface antigens were used: TcR1-FITC (clone GL3) from AbD Serotec and CD19-PE-Cy5.5 (clone 6D5), CD3-APC (clone 145-2C11), CD45-FITC (clone 30-F11), CD16/32-PE (clone 93) and CD14-FITC (clone Sa2-8) from eBioscience. Before the flow cytometry, the isolated lymphocytes were incubated with the appropriate antibodies for 30 min, washed twice in PBS and analyzed by FACSCalibur™ (BD Biosciences) equipped with a 488 nm argon-ion laser and a 633 nm diode laser. At least 10^5 ^cells were analyzed and data analyses of gated lymphocytes positive for CD45 were performed using CELLQuest™ Pro software (BD Biosciences). γδ T-lymphocytes were identified in a single TcR-specific staining. CD19-positive B-lymphocytes and CD3-positive T-lymphocytes, and CD4 and CD8 Th- and Tc-lymphocytes, were each characterized by separate two-colour analysis. Finally, the CD14 and CD16 positive cells out of CD3 and CD19 double negative were quantified using a four-colour analysis.

### Real time PCR

Total RNA was extracted from caecal wall samples using the RNeasy Lipid Tissue Kit (Qiagen). Resulting RNA was eluted with 50 μl RNase-free water and used immediately in reverse transcription using M-MLV reverse transcriptase (Invitrogen) and oligo-T primers. The resulting cDNA was purified by the QiaPrep PCR Purification kit (Qiagen) and used as a template for quantitative PCR. mRNA expression rates of TNFα, IL-12p40, IL-18, IFNγ and iNOS were determined using the QuantiTect™ SYBR^® ^Green RT-PCR Kit (Qiagen) with β-actin mRNA as a reference. Primers used for the RT-PCR are listed in Table 4. The threshold cycle values (Ct) of gene of interest were first normalised to the Ct value of actin reference mRNA (ΔCt) and the normalised mRNA levels were calculated as 2^(-ΔCt)^. The normalised mRNA levels of a particular cytokine were then used for t-test comparisons between the infected and non-infected animals and are also given in figures as "actin" units.

**Table 4:** List of primers used for the quantification of gene expression by real time RT PCR.

primer	sequence 5'-3'	length (bp)	Reference
TNFαFor	CATCTTCTCAAAATTCGAGTGACAA	175	[34]
TNFαRev	TGGGAGTAGACAAGGTACAACCC		
IL-12p40For	GGAAGCACGGCAGCAGAATA	180	[34]
IL-12p40Rev	AACTTGAGGGAGAAGTAGGAATGG		
IL-18For	CAGGCCTGACATCTTCTGCAA	105	[34]
IL-18Rev	TCTGACATGGCAGCCATTGT		
IFNγFor	AACAGCAAGGCGAAAAAGGA	92	this study
IFNγRev	GTGGACCACTCGGATGAGC		
iNOSFor	CAGCTGGGCTGTACAAACCTT	95	[34]
iNOSRev	CATTGGAAGTGAAGCGTTTCG		
β-actinFor	CTTTGCAGCTCCTTCGTTG	150	this study
β-actinRev	ACGATGGAGGGGAATACAGC		

### Statistical analysis

Data were evaluated by parametric two-sample, equal variance, t-test and non-parametric Mann-Whitney test comparing the experimental groups either to the non-infected control mice or to the mice infected with the wild type *S*. Enteritidis. Survival rates were analysed using Logrank test. Minor differences in the results of t-test and Mann-Whitney test were recorded only during the analysis of data presented in Table 2 in CD4 and CD8 T-lymphocytes, and γδ T-lymphocytes. All remaining significant differences were identically confirmed by both these tests and in figures we therefore refer only to the results of the t-test. In all the tables and figures, the average values of the individual animals ± standard deviation are shown. In some of the data analyses we clustered the mutants according to the presence of SPI-2 in their genome. All the statistical calculations have been performed using Prisma statistical software.

## Authors' contributions

DK and AS constructed the SPI mutants, FS and HH were responsible for the animal experiments. VK performed the histology and JV determined the cytokine expression by RT PCR. MF and PO were responsible for the flow cytometry. IR designed the studies and wrote the manuscript. All authors read and approved the final manuscript.
